# A Mutation in *Mtap2* Is Associated with Arrest of Mammalian Spermatocytes before the First Meiotic Division

**DOI:** 10.3390/genes2010021

**Published:** 2011-01-10

**Authors:** Fengyun Sun, Mary Ann Handel

**Affiliations:** The Jackson Laboratory, 600 Main Street, Bar Harbor, ME 04609, USA; E-Mail: fengyun.sun@jax.org

**Keywords:** meiosis, spermatogenesis, microtubule-associated protein

## Abstract

In spite of evolutionary conservation of meiosis, many of the genes that control mammalian meiosis are still unknown. We report here that the ENU-induced *repro4* mutation, identified in a screen to uncover genes that control mouse meiosis, causes failure of spermatocytes to exit meiotic prophase I via the G2/MI transition. Major events of meiotic prophase I occurred normally in affected spermatocytes and known regulators of the meiotic G2/MI transition were present and functional. Deep sequencing of mutant DNA revealed a mutation located in an intron of the *Mtap2* gene, encoding microtubule-associated protein 2, and levels of *Mtap2* transcript were reduced in mutant testes. This evidence implicates MTAP2 as required directly or indirectly for completion of meiosis and normal spermatogenesis in mammals.

## 1. Introduction

In mammals, meiosis is comprised of two consecutive cell divisions following one round of DNA replication to give rise to four haploid secondary spermatocytes. In spite of evolutionary conservation of the process, many of the genes that control mammalian meiosis are still unknown and the degree of sexual dimorphism in meiosis remains a puzzle [1]. Identification of genes involved in meiosis can provide insight for better understanding of “maturation arrest” male infertility phenotypes. In humans, about half of infertility is related to male reproductive function; however, the etiology of much of this is not known [2–4].

Our work focuses on one of the least understood steps in meiosis I, which is the exit from the extended prophase I and onset of the division phase, also known as the meiotic prophase I to metaphase I, or G2/MI, transition. This is regulated in part by MPF (metaphase-promoting factor), comprised of a catalytic subunit, cyclin dependent kinase 1 (CDK1), and a regulatory subunit, cyclin B1 (CCNB1) [5–7]. The association of CDK1 with CCNB1 represents a key step and is regulated by the chaperone protein HSPA2 [7–9]. Together, these and other as yet unknown regulators bring about progressive changes in the nucleus and cytoskeleton, including desynapsis, chromosome condensation, disassembly of the nucleolus, and nuclear envelope breakdown [5,10–12]. Consequently, it is believed that the cytoskeleton may play roles in these events, and, interestingly, cytoskeletal elements, such as actin [13,14] and microtubule-associated proteins (MAPs) [15–17] have been reported in nuclei of spermatocytes. Furthermore, MAPs have been shown to mediate the association of MPF with microtubules [18,19]. Thus, function of microtubules and MAPs in meiotic cell cycle events is possible, but there has not been genetic evidence that this is the case.

Here we used an unbiased forward genetics strategy [20–22] to produce phenotypes of meiotic arrest and aberrant exit from meiotic prophase I in mouse spermatocytes. These phenotypes were found in homozygous *repro4*/*repro4* mutants (here designated as *repro4* mutants). A mutation in an intron of the *Mtap2* gene, encoding microtubule-associated protein 2 protein, MTAP2, was identified in *repro4* mutant DNA. Levels of the *Mtap2* transcript are reduced in *repro4* mutant testes. The meiosis arrest phenotype associated with the *Mtap2* mutation in *repro4* mutant mice provides the first genetic evidence that a microtubule-associated protein functions, either directly or indirectly, in meiotic prophase I exit during mammalian spermatogenesis.

## 2. Results and Discussion

The *repro4* mutant line was produced by the NIH-funded Reproductive Genomics program at The Jackson Laboratory [22]. The mutation was induced on a C57BL/6J (herein, B6) background and ENU-treated males were subsequently mated to C3HeB/FeJ (herein, C3H) females to generate G1 males, which were mated with C3H females to produce G2 offspring. G2 females were backcrossed to their G1 fathers to generate G3 progeny, which were tested for fertility and used to generate mice for initial gene mapping. The *repro4* mutation segregates as a simple recessive Mendelian trait causing male-limited infertility.

### 2.1. The repro4 Mutation Causes Meiotic Prophase I Arrest in Males

The *repro4* mutant males were overtly normal except that testis weights of adult mutants were about one third that of wild type (WT) littermates (Table 1) and only a few epididymal sperm were recovered from adult *repro4* mutants. Nonetheless, the *repro4* mutant males produced plugs in female mice, suggesting normal mating behavior.

**Table 1.** Mean testis weights of WT and *repro4* mutant mice.

**Genotype**	***n***	**Age (weeks)**	**Body weight (g)**	**Testis weight (mg)**
WT	5	8	27.18 ± 1.30	84.18 ± 2.10
*repro4* mutant	7	8	28.7 ± 2.67	35.45 ± 1.52

Data are presented as mean±SEM.

**Figure 1:**
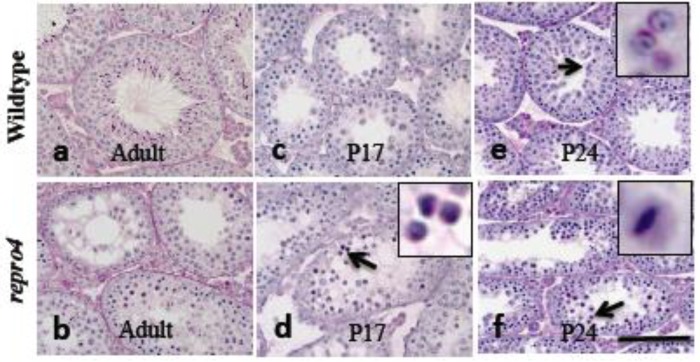
Spermatogenesis is arrested predominantly during prophase of meiosis I in *repro4* mutants. PAS-stained testis sections from *repro4* mutants and WT littermate controls reveal that spermatogonia, spermatocytes and spermatids are present in testes of adult WT mice (**a**), whereas in *repro4* mutant testes, most tubules contain only spermatogonia and spermatocytes (**b**). By P17, during the first wave of spermatogenesis, WT and *repro4* mutant testis tubules were populated with spermatocytes, but *repro4* mutants (**d**) exhibited more spermatocytes with highly condensed chromatin (arrow and inset) than did WT testes (**c**). By P24, round spermatids were present in WT testes (**e**, arrow and inset), but *repro4* mutant testis tubules contained primarily prophase and a few metaphase spermatocytes (**f**, arrow and inset). Scale bar for **a–f** = 100 μm.

In adult WT males, all stages of spermatogenesis—spermatogonia, spermatocytes, and spermatids—were observed in seminiferous tubules (Figure 1a). However in *repro4* mutants, although spermatogonia and spermatocytes were observed, only a few tubules with spermatids were observed (Figure 1b), suggesting arrest of spermatogenesis in prophase of meiosis I. To determine whether the mutation influences spermatogonial proliferation, antibody to GCNA1 (germ cell nuclear antigen1) [23] was used to label the testis sections from postnatal day 8, 12, 17 mice; there were no differences in the number of spermatogonia between the mutants and their WT littermates (data not shown). Together, these observations reveal that the *repro4* mutation does not severely impact spermatogonial proliferation, but causes arrest of spermatogenesis during prophase I.

To more precisely define the stage and nature of the spermatogenesis arrest phenotype of the *repro4* mutation, we analyzed the first wave spermatogenesis in detail by histology and immunofluorescent detection of markers of meiotic progression. Histology of testes at postnatal day 8 (P8), P11 and P14 showed no obvious differences in either morphology or histological relationships of various cell types or germ cell number between *repro4* mutants and WT littermates (data not shown). By P17, spermatogonia and spermatocytes were observed in both WT and *repro4* mutant testes. However, more spermatocytes with condensed chromatin, an indication of apoptosis, were observed in *repro4* mutant testes than in testes of WT littermates (Figure 1c and d, arrow and inset in d). By P24, in marked contrast to WT littermates (Figure 1e, arrow and inset), very few round spermatids were seen in *repro4* mutant testes (Figure 1f). In *repro4* mutant testes, germ cells in most seminiferous tubules were at stages of prophase I, although metaphase spermatocytes were observed in a few tubules (Figure 1f, arrow and inset). Thus, in *repro4* mutant testes, progress of spermatogenesis appeared to be arrested during prophase of meiosis I, although a few spermatocytes reached more advanced stages.

**Figure 2:**
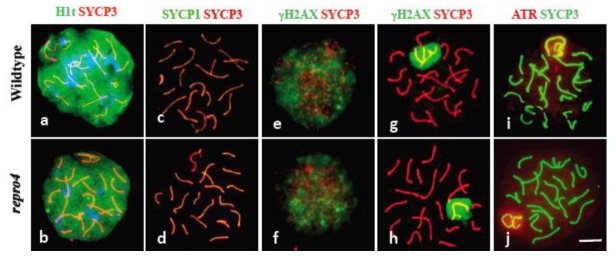
Essential events in the prophase of meiosis I appear normal in *repro4* mutant testes. By P17, both WT (**a**) and *repro4* mutant (**b**) spermatocytes have accumulated histone H1t (green), a marker of the mid-pachytene stage, seen in the chromatin surrounding the SYCP3 axes (red) of the synaptonemal complexes (SC). The extent of synapsis, reflected by the patterns of SYCP1 (green), a central element of the SC, and SYCP3 (red), a lateral element of the SC, was indistinguishable between WT (**c**) and *repro4* mutant (**d**) spermatocytes. The pattern of γH2AX labeling, a marker of DNA double strand break/repair, was also similar between WT (**e** and **g)** and *repro4* mutant (**f** and **h**) spermatocytes in early prophase (**e** and **f**) and in the mid-to late pachytene stage (**g** and **h**). The labeling pattern of the DNA damage checkpoint protein ATR at pachytene stage appears normal in *repro4* mutant spermatocytes (**j**) compared to WT controls (**i**). Scale bar = 10 μm.

These observations of histology sections suggested spermatocytes in *repro4* mutants reached the pachytene substage of meiotic prophase I. To confirm this, surface-spread spermatocyte chromatin was analyzed for presence of histone H1t, a marker of the mid-pachytene stage. As shown in Figure 2a, b, histone H1t was detected with appropriate nuclear localization in *repro4* mutant spermatocytes, confirming that germ cell development in *repro4* mutants reaches at least the mid-pachytene stage before arrest. Thus, although *repro4* mutant spermatocytes reach the stage when competence to undergo the meiotic prophase I to metaphase I transition is normally acquired [5,6], the *repro4* mutant spermatocytes failed to undergo this transition successfully *in vivo*.

Because of this arrest, we assessed whether *repro4* mutant spermatocytes undergo apoptosis. TUNEL analysis revealed a significant increase (*p* < 0.05) of the number of apoptotic cells per tubule in *repro4* mutant testes compared to littermate controls (Table 2). An increase of the frequency of tubules with apoptotic cells in *repro4* mutant testes was also observed compared to that in WT controls at P17, although the difference was not significant (Table 2). The apoptotic cells were detected luminal from the basement membrane of the seminiferous tubules, not apposed to the basement membrane. This location suggests that they were spermatocytes, but not spermatogonia or Sertoli cells. This inference was bolstered by GCNA staining, which revealed no difference in the number of spermatogonia between the *repro4* mutant and WT testes (see above). Moreover, an increase in apoptosis was also observed by flow cytometry after labeling apoptotic cells with annexin V (data not shown). Thus germ cells in *repro4* mutant testes undergo apoptosis during meiotic prophase I.

**Table 2.** Analysis of apoptosis in *repro4* mutants.

	**WT, P17**	***repro4*, P17**	**WT, P21**	***repro4*, P21**
Apoptotic tubules/total tubules	0.34 ± 0.05	0.43 ± 0.03	0.38 ± 0.02	0.47 ± 0.07
Apoptotic cells/tubule	0.69 ± 0.11 *	2.19 ± 0.38 *	0.85 ± 0.13 *	2.17 ± 0.36 *

Data are presented as mean ± SEM.

* *p* < 0.05. P, postnatal day; WT, wild type; *repro4*, *repro4* mutant. Three animals of each group and at least 40 tubules of each animal were analyzed.

### 2.2. Major Cytological Events of Prophase I in repro4 Mutants Are Apparently Normal

To determine if the failure of *repro4* mutant spermatocytes to enter the meiotic division phase was due to deficiencies of key events of meiotic prophase I, we assessed markers of these events, including synaptonemal complex formation, meiotic DNA double-strand break formation and repair, and localization of DNA damage checkpoint proteins. As shown in Figure 2c, d, the labeling patterns for SYCP3 and SYCP1 proteins, components of the lateral element and central element, respectively, of the synaptonemal complex, in *repro4* mutant spermatocytes was indistinguishable from that in WT littermate spermatocytes. Thus synapsis appears to be substantially normal in *repro4* mutant germ cells, and, indeed, even diplotene spermatocytes were found among *repro4* mutant spermatocytes, as determined from the labeling pattern of SYCP3 (data not shown). During meiosis, DNA double strand breaks (DSBs) occur in early prophase and are then repaired as germ cells reach the pachytene stage. The localization of phosphorylated histone H2AFX (γH2AX), a marker of DNA DSBs, was identical in leptonema/zygonema and pachynema of both *repro4* mutants and WT littermates (Figure 2e-h), suggesting that as in WT spermatocytes, DSBs occurred and were repaired in the *repro4* mutant spermatocytes. Ataxia telangiectasia and Rad3-related protein (ATR) is involved in sensing DNA damage and activating the DNA damage checkpoint, leading to cell cycle arrest [24]. To verify that DSBs were repaired in *repro4* mutant spermatocytes and confirm that the arrest of *repro4* mutant spermatocytes was not due to DNA damage, ATR labeling was performed. Consistent with γH2AX labeling, the labeling pattern of ATR was indistinguishable between *repro4* mutant spermatocytes and littermate controls (Figure 2i, j). Likewise, condensation of the X and Y chromosomes to form the sex body occurred normally in *repro4* mutant spermatocytes, as indicated by the accumulation of γH2AX in the sex body, marking the condensed X and Y chromosomes (Figure 2g, h).

**Figure 3:**
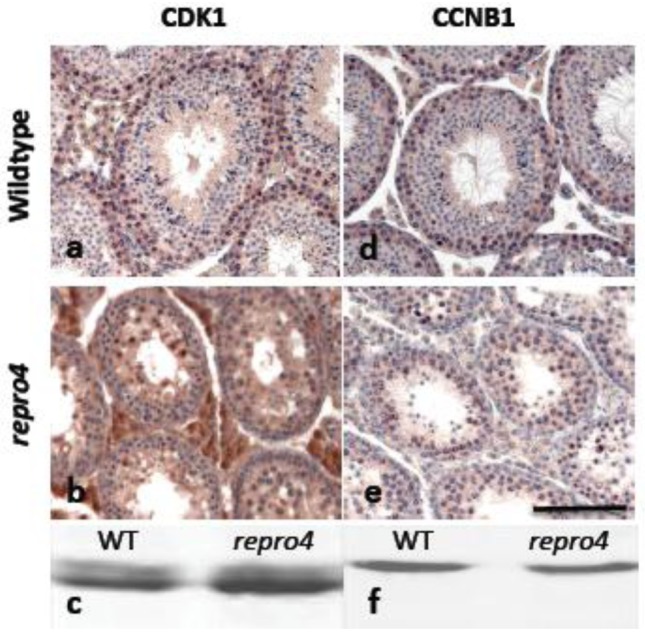
Protein components of MPF are present in *repro4* mutant spermatocytes. Both immunohistochemistry (**a**, **b**, **d** and **e**) and western blotting (**c** and **f**) revealed that CDK1 (**a**, **b** and **c**) and CCNB1 (**d**, **e** and **f**) were present in both WT and *repro4* mutant spermatocytes. WT, wild type; *repro4*, *repro4* mutant. Scale bar = 100 μm.

These observations suggest that DNA damage/repair, and meiotic chromosome pairing and synapsis in *repro4* mutant germ cells are cytologically normal, and may not be causative for the failure of mutant spermatocytes to undergo the transition from meiotic prophase I to metaphase I. We thus hypothesized that the cell cycle machinery may not be functional in *repro4* mutant spermatocytes. The universal regulator of prophase to metaphase transition in both mitosis and meiosis is MPF, composed of a regulatory subunit of CCNB1 and a catalytic subunit of CDK1 [6,25,26]. Immunohistochemistry and western blotting were used to assess the presence of CCNB1 and CDK1 in *repro4* mutant spermatocytes. As shown in Figure 3, CDK1 and CCNB1 were observed in *repro4* mutant spermatocytes as in WT spermatocytes.

**Figure 4:**
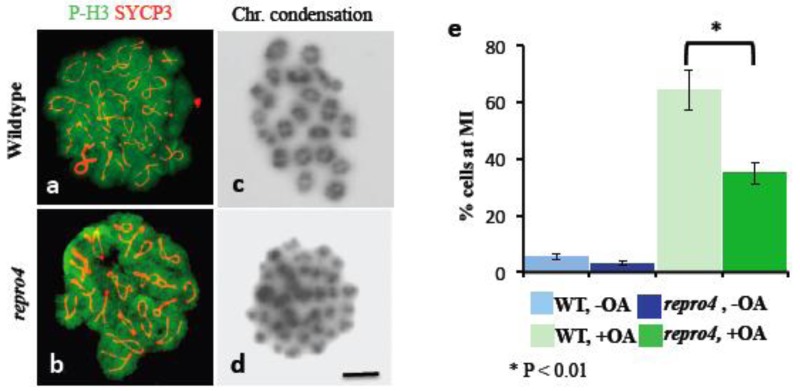
The OA-induced G2/MI transition is not normal in *repro4* mutant spermatocytes. After treatment of spermatocytes with okadaic acid *in vitro*, phosphorylation of histone H3 (green) around the SYCP3 axes of the synaptonemal complex (red) occurred in both WT (**a**) and *repro4* mutant (**b**) spermatocytes. (**c**) shows the normal chromosome configuration after spermatocytes were treated with OA *in vitro*, and (**d**) shows a representative chromosome configuration after *repro4* mutant spermatocytes were treated with OA *in vitro*. (**e**) presents these observations graphically and demonstrates that about 65% WT spermatocytes formed condensed and individualized bivalent chromosomes, whereas significantly fewer (*p* < 0.01, about 30%) *repro4* mutant spermatocytes formed condensed and individualized bivalent chromosomes. This experiment was performed 3 times, with at least 200 spermatocytes counted per experiment. 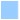
WT, −OA: wild type spermatocytes without OA treatment; 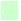
WT, +OA: wild type spermatocytes with OA treatment; 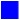
*repro4*, −OA: *repro4* mutant spermatocytes without OA treatment; 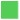
*repro4*, +OA: *repro4* mutant spermatocytes with OA treatment. Scale bar for **a–d** = 10 μm.

To determine competence of *repro4* mutant spermatocytes to undergo the G2/MI transition, we treated *repro4* mutant and WT spermatocytes *in vitro* with okadaic acid (OA), an inhibitor of protein phosphatase 1 and 2A. OA induces MPF activation and the meiotic prophase I to metaphase I transition *in vitro* [5]. Progress of the G2/MI transition was evaluated by two parameters: The formation of fully condensed and individualized metaphase I bivalent chromosomes and phosphorylation of histone H3. Histone H3 phosphorylation occurred in *repro4* mutant spermatocytes as in WT littermate spermatocytes (Figure 4a, b), suggesting that aurora kinase B, reported to be involved in meiotic prophase I to metaphase I transition [6], was functional in the *repro4* mutant spermatocytes. However, although chromatin condensation was observed in OA-treated *repro4* mutant spermatocytes, unlike chromosomes of WT spermatocytes (Figure 4c), many *repro4* mutant spermatocytes could not form individualized bivalent metaphase I chromosomes after OA treatment (Figure 4d). This suggests the failure of G2/MI chromatin remodeling events in *repro4* mutant spermatocytes *in vitro* as well as *in vivo*.

Taken together, the results presented in Figures 3 and 4 indicate that major events of prophase of meiosis I occur in *repro4* mutant spermatocytes, and at least some of the cell cycle machinery required for the G2/MI transition is functional. However, *repro4* mutant spermatocytes are incapable of executing the meiotic G2/MI transition normally *in vivo* and *in vitro*. These observations suggest that some as yet unknown factor must be required for regulation of this transition and chromatin remodeling and be absent or defective in *repro4* mutant testes.

### 2.3. Sequencing of repro4 Mutant DNA Reveals a T to A Transversion Mutation in Mtap2

**Figure 5:**
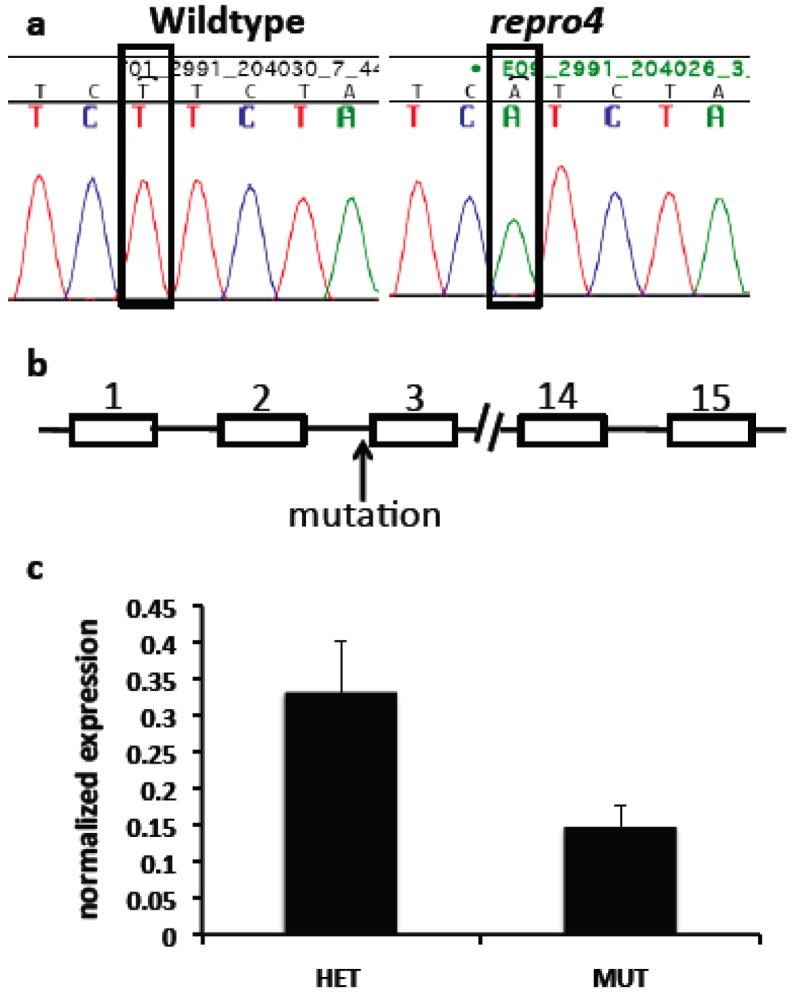
A mutation was identified in the *Mtap2* gene in *repro4* mutant DNA. The T to A transversion is marked by an open box (**a**). The mutation is located between exon 2 and exon 3 of *Mtap2* (**b**). Quantitative RT-PCR was used to assess levels of *Mtap2* transcript in P8 heterozygote and *repro4* mutant testes (**c**).

A genome scan of DNA from affected and unaffected mice in the *repro4* family for polymorphic markers on each autosomal chromosome revealed the *repro4* mutation on Chr 1 between *D1Mit303* and *D1Mit415*, a region of about 25.4 Mb. Fine mapping within this region was conducted. Homozygous *repro4* mutant females were crossed to CAST/EiJ (herein CAST) males, and the F1 offspring were intercrossed to produce F2 individuals that were subjected to phenotype analysis and typed for polymorphic markers within the region. Additionally, recombinants obtained from the C3H-*repro4* maintenance line were also used to narrow the candidate region. The critical region for the *repro4* mutation was an interval of about 2.1 Mb between *D1Mit325* and *D1Mit21* (Chr. 1 64901285–66992999 bp), containing 38 genes, of which 28 genes are known and 10 genes are novel [27]. The exons and exon-intron boundaries of 22 genes were sequenced and no mutation was found. Next targeted resequencing of *repro4* mutant DNA in a region of Chr. 1 (64,835,695–66,992,999 bp) was conducted. *repro4* mutant DNA from this region was enriched using a custom NimbleGen capture array and sequencing was performed on Illumina Genome Analyzer 2. Bioinformatic analysis revealed no mutations within exons, consistent with our sequencing results. One mutation, in an intron of the *Mtap2* (microtubule-associated protein 2) gene that is located between exons 2 and 3 in ENSMUST00000114015 and ENSMUST00000114017 transcripts, was confirmed by sequencing *repro4* mutant and B6 tail DNA (Figure 5a, b). This was a T to A transversion (Figure 5a). Subsequently, expression of *Mtap2* in WT and *repro4* mutant testes was examined. There are 9 protein-coding transcripts for *Mtap2* [27]. The gene expression assay used covered 5 *Mtap2* transcripts: ENSMUST00000114015, ENSMUST00000024639, ENSMUST00000114017, ENSMUST00000114013, ENSMUST00000114018. The remaining protein coding transcripts were not analyzed because they are at least 110 kb distant from the mutation. Quantitative RT-PCR showed that the transcript level of *Mtap2* was more than 50% less in *repro4* mutant testes than in heterozygote littermate controls at postnatal day 8 (Figure 5c). A western blot analysis was performed to determine if MTAP2 protein was present; there were no major differences between the wild type and the *repro4* mutant (data not shown). The antibody used for this analysis detects numerous MAPs, but we do not know if it detects all isoforms expressed in testes, or whatever isoform(s) might be affected by the *repro4* mutation. Nonetheless, this result suggests the possibility that, despite reduced *Mtap2* transcript, the protein amounts may not be significantly reduced.

The *Mtap2* locus in the mouse is complex, and up to 22 different mRNA transcripts can be produced. The transcripts differ by truncation of the 5′-end, truncation of the 3′-end, presence or absence of 9 cassette exons, overlapping exons with different boundaries, alternative splicing or retention of one intron. Additionally, there are four probable alternative promoters. Of the predicted transcripts, 19 spliced and 2 unspliced mRNAs putatively encode a functional protein (20 different isoforms). The remaining mRNA variants (unspliced) appear not to encode protein [28]. Furthermore, 1025 bp of this gene are antisense to putative spliced predicted gene Tahorora, raising the possibility of regulated alternative expression. Thus there is considerable molecular complexity, which may be reflected in biological complexity. In the literature, the encoded MTAP2 proteins are referred to as MAP2s (microtubule-associated protein 2). The MAP2 protein identified in humans and rats consists of alternatively spliced isoforms in two groups: high-molecular weight MAP2, including MAP2A and MAP2B, and low-molecular weight MAP2 including MAP2C and MAP2D with different spatiotemporal expression patterns in neurons [29–32]. Indeed, all studies of MAP2 are related to its function in neurons. However, the so-called “neuron-specific” MAP2 is also present both in somatic cells and germ cells of the rat testis [15,16]. The predominant MAP species in the rat testis is proposed to be the MAP2C protein. In addition to cytoplasmic localization, MAP2 was also found in nuclei of Sertoli cells, as well as pachytene and diplotene spermatocytes in rat testes, perhaps even co-localizing with the synaptonemal complex as MAP1B [17]. Based on these data, the mouse MTAP2 protein could be present in testes at the right time and in the right place to play roles in the meiotic prophase I to metaphase I transition. Our finding that a mutation in the *Mtap2* gene in the mouse is associated with prophase arrest of meiosis I is consistent with this hypothesis.

How might the MTAP2 protein act in meiotic progress? MTAP2 is a microtubule stabilizing protein [29]. It is possible that the meiotic prophase arrest phenotype of *repro4* mutants is a result of destabilization of microtubules, due to diminished *Mtap2* expression. Indeed, the microtubule-destabilizing drug, colchicine, causes arrest of cell cycle both in mitosis and meiosis, as well as failed migration of elongated spermatids toward the tubule lumen and abnormal morphogenesis of the sperm head and flagellum [33–37]. Thus microtubule dynamics can be inferred to be an important regulatory mechanism in spermatogenesis. It has been demonstrated that MAPs can modulate the microtubule dynamics at extremely low concentrations [38], and possibly diminished, but not ablated, expression of *Mtap2* could impact on testicular microtubule dynamics. If this is occurring in *repro4* mutant testes, the implication is that microtubule dynamics may directly or indirectly influence meiotic progression from prophase I to the division phase, a previously unsuspected mechanism. More broadly, the involvement of MTAP2 in meiosis, implied by the *repro4* mutant phenotype, is consistent with emerging evidence that motor proteins, which often use cytoskeleton for movement, are not only present in nuclei, but also play significant roles in chromosome condensation and segregation [39,40]. However, it is not known whether the meiotic arrest phenotype is a direct or indirect effect of the *Mtap2* mutation. Thus, although this report provides novel data implicating *Mtap2* in meiotic cell cycle regulation, more work is required to resolve the mechanism of the *Mtap2* mutation in regulation of the meiotic G2/MI transition.

## 3. Experimental Section

### 3.1. Animals

All mice were bred and raised in the research colony of the authors at The Jackson Laboratory. Protocols for their care and use were approved by the Institutional Animal Care and Use Committee (IACUC) of The Jackson Laboratory. All the *repro4* mutant mice used for biological study were from a congenic line maintaining the *repro4* mutation on a C3H background.

### 3.2. Mutagenesis, Genetic Fine Mapping and Sequencing

Mutagenesis of adult male C57BL/6J (B6) mice were carried out with injection of ethylnitrosourea (ENU) as previously described [41]. The mice were subsequently crossed with C3HeB/FeJ (C3H) females to create G1 male founders of pedigrees. G1 males were mated to C3H females to produce G2 offspring. G2 daughters were backcrossed to the G1 father to generate the G3 population. The G3 offspring were tested for fertility phenotype [42].

DNA was extracted from a piece of tail of infertile G3 animals in the *repro4* pedigree, and a genome scan was carried out by the Fine Mapping Service of The Jackson Laboratory using microsatellite markers polymorphic between B6 and C3H. Linkage analysis mapped the mutation to chromosome 1. For the genetic fine mapping, *repro4* mutant females were crossed to strain CAST/EiJ males to take advantage of greater polymorphism. Genotyping was done by PCR amplification of tail DNA. The critical region was mapped to chromosome 1 between *D1Mit325* and *D1Mit21*, about 2.1 Mb.

For sequencing, DNA was obtained from extraction of tails *of repro4* mutant, *repro4* heterozygous and B6 mice. After PCR amplification, products were run on 2% SeaKem^®^ LE Agarose gel (Lonza). Suitable bands were then cut and DNA was extracted using QIAquick^®^ Gel Extraction Kit (Qiagen). DNA sequencing was performed using standard methods by the DNA Sequencing Service of The Jackson Laboratory. Only exons and intron-exon boundaries were sequenced.

For deep sequencing, DNA was isolated by the DNA Resource Service of The Jackson Laboratory. DNA samples were reextracted once with phenol-chloroform; the 260/280 ratio was about 2.0. DNA samples were enriched using a custom NimbleGen capture array and single lane sequencing on Illumina Genome Analyzer 2 was performed.

### 3.3. Histology and Immunohistochemistry

Testes were fixed in Bouin's or 4% paraformaldehyde in PBS overnight and paraffin-embedded. 5 μm sections were cut, dewaxed and stained with Periodic Acid Schiff (PAS). For immunohistochemistry, the sections were blocked with 5% goat serum in PBS at room temperature for 30 min. Primary antibodies were applied to the sections and incubated at 37 °C for 1 h. The antibodies used included rabbit antibody to CDC2 (CDK1) (Santa Cruz) at 1:50 and mouse CCNB1 antibody (Abcam) at 1:400. After two washes with PBS, the color reactions were performed according to SuperPicTure^TM^ polymer detection Kit (Zymed). Briefly, the sections were incubated with HRP polymer conjugate (reagent A) at 37 °C for 10 min, followed by two washes of PBS. AEC single solution chromogen (reagent B) was added to the sections, and the sections were then incubated at room temperature for 12 min. After rinsing with distilled water, Hematoxylin (Sigma) was used for counterstaining. Clearmount (Zymed) was used as mounting solution. Images were acquired with a Leica DMRXE microscope coupled with a DFC 300FX R1 CCD camera.

### 3.4. Germ Cell Isolation and Spermatocyte Enrichment

Mice at suitable ages were killed by cervical dislocation. Testes were removed, detunicated and digested in 0.5 mg/mL collagenase (Sigma) in Krebs-Ringer bicarbonate solution (KRB) (120.1 mM NaCl, 4.8 mM KCl, 25.2 mM NaHCO_3_, 1.2 mM KH_2_PO_4_, 1.2 mM MgSO_4_·7H_2_O, 1.3 mM CaCl_2_, 11 mM glucose, 1 X essential amino acids, 1 X nonessential amino acids) at 32 °C for 20 min, followed by digestion in 0.5 mg/mL trypsin (Sigma) containing 20 mg/mL DNase I in KRB at 32 °C for 13 min. After digestion the cell suspension was filtered through an 80-μm mesh filter and washed three times in KRB. Germ cells were then processed as described below.

### 3.5. Okadaic Acid-Induced Meiotic Prophase I to Metaphase I (G2/MI) Transition

Enriched germ cells were placed in culture medium as previously reported [43] and incubated at 32 °C for 4 hours for recovery. The cells were then induced to undergo the G2/MI transition by the addition of OA (CalBiochem), dissolved at 300 μM in ethanol and used at 4 μM in the culture, while control cells were treated with the same volume of ethanol. After 4 h culture, the cells were collected for air-dried chromosome preparation.

### 3.6. Surface-Spread Chromatin Preparation

Spermatocytes were collected by centrifugation, surface-spread in wells of multi-spot microscope slides (Thermo) and fixed following the procedure previously described [44,45]. Prior to antibody labeling, slides were washed three times in washing/blocking buffer (0.3% BSA, 1% goat serum in phosphate-buffered saline, pH 7.4); the second wash included 0.05% Triton-X 100. After draining, the slides were incubated overnight with primary antibodies. Primary antibodies used were rabbit anti-SYCP1 (Novus) at 1:100; rabbit anti-SYCP3 (Novus) at 1:100; guinea pig anti-H1t [5] at 1:500; rabbit anti-γH2AX (Millipore) at 1:200; rat anti-SYCP3 [46] at 1:1000; rabbit anti-ATR (Santa Cruz) at 1:100. Secondary antibodies against rabbit, rat, or guinea pig IgG and conjugated with Alexa 594 or 488 (Molecular Probes) were used at 1:500 dilution. Images were acquired with a Leica DMRXE epifluorescence microscope equipped with a 100X plan-neofluar oil-immersion objective lens and a triple filter (set no. 61000V2 BS&M, Chroma Technology for simultaneous visualization of green (Alexa 488), red (Alexa 594), yellow (Alexa 488 + Alexa 594) and blue (DAPI) fluorescence). The microscope was linked to a Micromax cooled CCD camera (RS Princeton Instrument), a high-speed shutter driven by a Sutter Lambda 10-2 (Sutter Instrument) and Metamorph software (Universal Imaging Corporation) to capture the images.

Chromosome condensation was evaluated from air-dried chromosome preparations [47] from cultured spermatocytes collected by centrifugation and washed in 2.2% sodium citrate. The air-dried chromosome preparations were stained with Gurr Giemsa (Invitrogen). G2/MI stages, from pachynema to MI were scored using an Olympus microscope with a 40× plan objective and 10× ocular lenses, and images were captured to Adobe Photoshop with a Hamamatsu C5810 color-chilled camera (Photonic System).

### 3.7. Western Blot Analysis

For western blot analyses, total protein was extracted from testes of P21 mice using RIPA buffer (Santa Cruz) containing protease inhibitor cocktail (Santa Cruz). Protein concentration was measured by the BCA method. 10 μg protein from each group was boiled for 3 min, and proteins were separated by electrophoresis using 10% SDS-PAGE. Proteins were transferred onto PVDF membrane (Millipore) based on the manufacturer's instruction. The blots were blocked with 5% dried milk in Tris-buffered saline with 0.1% Tween-20 overnight at 4 °C. Rabbit anti-CDC2 (CDK1) (Santa Cruz) at 1:200 and mouse anti-CCNB1 (Abcam) at 1:2000 were used. The blots were incubated for 2 h at room temperature, and then incubated with horseradish peroxidase-conjugated anti-IgG secondary antibodies made from mouse (Invitrogen) and rabbit (Invitrogen) for 1 h at room temperature. The proteins were detected using the ECL Plus Western Blotting Detection System (Amersham).

### 3.8. Quantitative RT-PCR

Total RNA was extracted from frozen testes of P8 mice using the RNeasy extraction kit with DNaseI treatment as described by the manufacturer (Qiagen). Samples were diluted to a concentration of 10 ng/μL, dispensed in single-use aliquots, and stored at −80 °C. cDNA was made by using QuantiTech Reverse Transcription Kit (Qiagen) and followed the manufacturer's instruction. Quantitative RT-PCR (qRT-PCR) was performed on the Applied Biosystems 7500 Real-Time PCR System. TaqMan^®^ gene expression assays Mm00485230_m1 for *Mtap2* and Hs99999901_s1 for 18 s control (Applied Biosystems) were used to determine the relative expression level of *Mtap2* according to the relative standard curve method.

## 4. Conclusions

The *repro4* mutant phenotype of meiotic arrest is associated with a mutation in an intron of the *Mtap2* gene, encoding a microtubule-associated protein. Although spermatocytes of *repro4* mutants appear to execute key events of meiotic prophase I normally, the meiotic prophase I to metaphase I transition did not occur, thus causing sterility, and suggesting that mutation of *MTAP2* might be associated also with cases of human infertility. The features of the *repro4* mutant phenotype raise the intriguing possibility of a hitherto unsuspected regulatory pathway governing the progress of meiosis, a pathway dependent on stability of microtubules and perhaps also implicating other nuclear motor proteins in meiotic chromosome condensation and segregation. The *Mtap2* gene and its encoded proteins exhibit considerable complexity and thus, further work should not only confirm mechanism for the effect of the detected mutation and the roles of MTAP2 protein in male germ cells, but also bring novel insights to meiotic chromosome dynamics.
